# Co-grazing of sheep and goats may not be an issue from a parasitological perspective

**DOI:** 10.2478/helm-2025-0030

**Published:** 2025-11-26

**Authors:** I. A. Kyriánová, I. Knížková, M. Ptáček, J. Nápravníková, O. Kopecký, T. Husák, J. Vadlejch

**Affiliations:** 1 Department of Environment, Faculty of Environment, Jan Evangelista Purkyně University, Pasteurova 3632/15, 400 96 Ústí nad Labem, Czech Republic; 2 Department of Zoology and Fisheries, Faculty of Agrobiology, Food and Natural Resources, Czech University of Life Sciences Prague, Kamýcká 129, 165 00 Prague – Suchdol, Czech Republic; 3 Department of Livestock Technology a Management, Institute of Animal Science, Přátelství 815, 104 00 Praha – Uhříněves, Czech Republic; 4 Department of Animal Science, Faculty of Agrobiology, Food and Natural Resources, Czech University of Life Sciences Prague, 129 Kamýcká, Praha – Suchdol 16500 Czech Republic; 5 Department of Biology and Ecology, Faculty of Science, Humanities and Education, Technical University of Liberec, Studentská 1402/2, Liberec 461 17, Czech Republic

**Keywords:** diseases, digestive tract, nematodes, pasture, small ruminants, mixed-species grazing

## Abstract

Gastrointestinal nematode (GIN) infections have a significant impact on the health and productivity of small ruminants, while data on mixed-species grazing systems in Central Europe are scarce. This study aimed to compare GIN species richness and infection intensity in co-grazed dairy sheep and goats under a conventional grazing system in the Czech Republic. Over a 12-month period, 210 goat and 196 sheep faecal samples were analyzed using the McMaster method, followed by larval culture. Both hosts harboured *Haemonchus contortus, Trichostrongylus/Teladorsagia* spp., and *Oesophagostomum columbianum*. Goats exhibited consistently higher egg shedding, with a mean peak egg count of 1240 EPG in June, whereas sheep reached a markedly lower peak of 620 EPG in February. In goats, *H. contortus* predominated year-round, while in sheep, *Trichostrongylus/Teladorsagia* spp. showed pronounced seasonal fluctuations, comprising up to 60% of larvae in autumn. Differences in infection intensity between species were statistically significant (U = 24 697.5, p < 0.001). These results support the hypothesis that co-grazing does not homogenise parasite burdens between host species and demonstrate species-specific seasonal infection dynamics. Such insights directly address the study’s aim of characterising species composition and infection intensity in co-grazed sheep and goats, providing an evidence-based basis for optimising sustainable parasite management in mixed grazing systems.

## Introduction

The welfare of ruminant farming is greatly affected by infections caused by gastrointestinal nematodes (GIN). Economic losses due to these parasites primarily manifest as reduced production rather than mortality, with estimates indicating that GIN infections contribute to an annual production cost of approximately €1.8 billion across the EU, including both production losses and treatment expenses (Charlier *et al*., 2020; Charlier *et al*., 2024). Therefore, infections caused by GINs are usually classified as a production disease (Marshall *et al*., 2012).

The most common GINs responsible for economic losses in small ruminants belong to the family Trichostrongylidae (trichostrongylids), particularly *Haemonchus contortus, Trichostrongylus* spp., and *Teladorsagia circumcincta* (O’Connor *et al*., 2006; Gilleard, 2013). The severity of infection depends on various factors, with the composition of the parasite species being one of the most crucial. Certain GIN species are more pathogenic to their hosts than others. In natural infections, hosts are usually infected by multiple GIN species simultaneously, whereas mono-infections are rare (Leignel & Cabaret 2001; Demeler *et al*. 2012).

Sheep and goats harbour identical GIN species (Hoste *et al*., 2008; Domke *et al*., 2013); however, some data suggest the possibility of different nematode strains between sheep and goats (Sutherland & Scott, 2010). Most existing data on host-parasite interactions have been collected from studies focusing solely on sheep, and their results have been applied to goats. It is assumed that, due to different evolutionary processes, sheep and goats have developed distinct strategies for controlling GIN infections – sheep rely primarily on their immune response. In contrast, goats are more dependent on grazing behavior (Houdijk *et al*., 2012). These two strategies rely on a balance between developing an immune response in sheep or a behavioural response that limits contact with infective larvae present in the vegetation in goats. In goats, avoidance of infective larvae associated with grazing is suspected to be high due to their grazing behaviour (Hoste *et al*., 2010). Observations conducted under natural grazing conditions in Australia and Scotland indicate that goats exhibit higher levels of parasitic infection than sheep when co-grazing, supporting the hypothesis of a weaker immune response in goats to GIN infections (Torres-Acosta & Hoste, 2008). Specifically, goats may take approximately 12 months to develop a complete immune reaction, whereas sheep typically develop immunity within six months (Vlassoff *et al*., 1999). Moreover, in sheep, the manifestations of nematode infection in adult females are much less severe compared to young animals, unlike goats, for which the response to parasites is not well described (Hoste *et al*., 2008). Furthermore, adult goats excrete significantly larger quantities of parasite eggs in their faeces compared to sheep. This difference is likely due to physiological and immunological variations between the species. Although extensive research has been conducted on GIN infections in sheep and goats worldwide, data on co-grazing systems under Central European climatic conditions remain scarce. Most available studies have focused on Southern or Northern European regions, where environmental factors significantly differ from those in Central Europe (Kyriánová *et al*., 2019; Ruiz-Huidobro *et al*., 2019). The present study aims to fill this knowledge gap by examining the GIN load and species richness of co-grazing dairy sheep and goats in the Czech Republic. Understanding these dynamics is crucial for optimizing parasite management strategies in mixed-species grazing systems in temperate climates.

This study tests the hypothesis that quantitative descriptors of GIN populations differ significantly between sheep and goats in co-grazing systems. The objectives of our study were: (i) to compare the intensity of GIN infections in sheep and goats; (ii) to identify the nematode species richness in sympatric sheep and goats.

## Materials and Methods

### Farm and animals

**Fig. 1.**
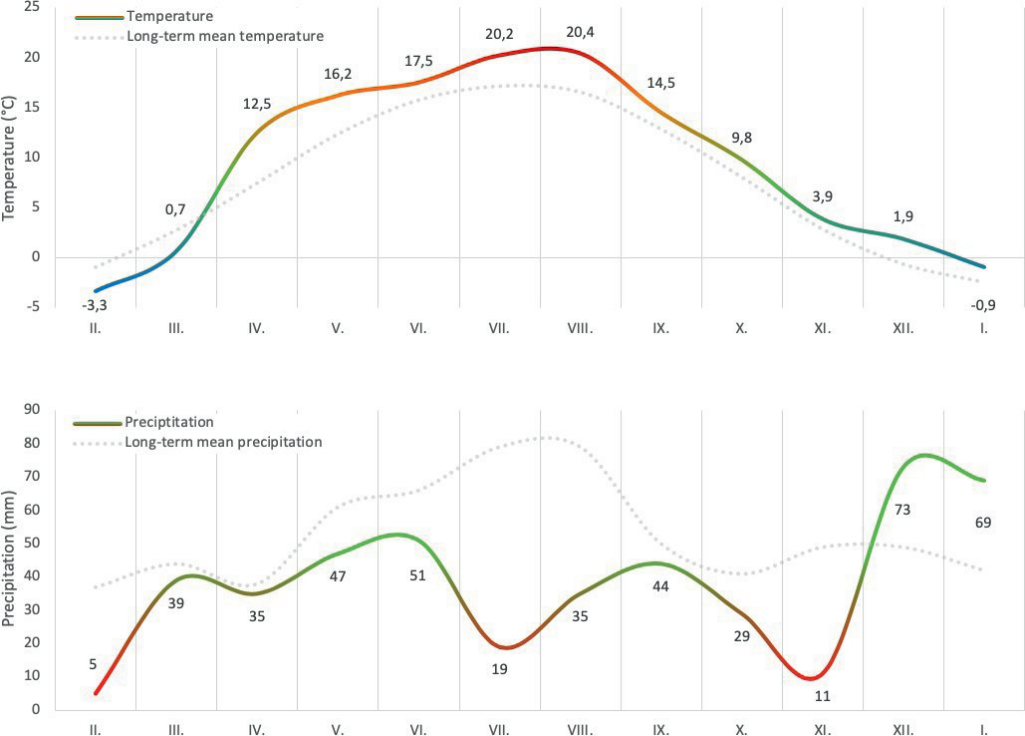
Average monthly temperature (°C) and precipitation (mm) recorded during the 12-month monitoring period. Data were obtained from a nearby meteorological station and reflect typical temperate climate conditions of the study area. Seasonal variation in temperature and rainfall may influence parasite development and transmission.

Our study was conducted over a continuous 12-month period, from February to January of the following year, on a conventional dairy farm in the northern Bohemia region, Czech Republic. The farm is situated at an altitude of 320 meters above sea level in a slightly warm, drier area with an average annual temperature of 9.1°C and an average annual rainfall of 670 mm. Basic meteorological data for the study period were obtained from the nearby meteorological station (see Fig. 1).

The base stock consisted of more than 200 animals, all of which were kept under identical farm conditions. About half of the flock comprised dairy goats (White Shorthaired and the Brown Shorthaired goat, and their crossbreds), while the other half consisted of dairy sheep (Laucane, East Friserian sheep, and their crossbreds). All animals had year-round unrestricted access to 45-hectare pastures, divided into smaller fenced areas with rotational grazing across four pastures. The rotation schedule was not fixed but depended on pasture condition and growth, with animals typically spending 2 – 3 weeks in each pasture.

In the barn, the animals had access to hay, water, and a partial mixed ration, while concentrates were provided during milking. The complete feeding regimen, including the botanical composition of pastures and other dietary components, was characterized in detail by Ptáček *et al*. (2021).

The farm processes milk into goat and sheep cheese, yoghurts, and other products, which it sells at local farmers’ markets. Animals were dried off based on milk production and udder status, but always at least eight weeks before the expected parturition. The mating period occurred from August to September, with the lambing, or kidding, period taking place from January to March. Lambs and goat kids were separated from their mothers immediately after birth and reared artificially on milk replacements. Granulates and hay were offered in the creeping pen from the 2^nd^ week of age. Lambs and kids were weaned after achieving 18 kg of their live weight.

Average milk yield during the lactation period was 3.04 kg/day in goats and 1.55 kg/day in sheep. Detailed monthly values for milk yield and basic composition parameters are provided in Table 1. These data were recorded as background herd characteristics; the present study did not assess milk composition or relate it to gastrointestinal nematode infection.

**Table 1.** Monthly milk production and composition in monitored goats and sheep during the lactation period. Values include milk yield (kg/day) and percentages of fat, protein, and lactose. Data were collected as part of the official milk recording system in the Czech Republic. Results represent the average performance of the sampled animals over five (goats) or four (sheep) monthly recordings.

Goats - monitoring performance check results				
	**milk (kg)**	**fat (%)**	**protein (%)**	**lactose (%)**
III.	2.9	4.00	3.15	4.56
IV.	3.0	2.69	2.72	4.18
V.	3.1	2.80	2.95	4.32
VI.	3.3	2.70	2.96	4.52
VII.	2.9	2.93	3.01	4.32
	mean	mean	mean	mean

Sheep - monitoring performance check results				
	**milk (kg)**	**fat (%)**	**protein (%)**	**lactose (%)**
III.	1.5	7.61	5.39	4.83
IV.	2.0	5.62	5.64	4.70
V.	1.3	7.03	6.03	4.72
VI.	1.4	7.27	6.42	4.68
	mean	mean	mean	mean

### Anthelmintic treatment regimen

Weaned animals and adult males were kept separately on pasture, with or without shelters, and with or without rotational grazing. The herd operated under a closed turnover policy, except for the introduction of new sires to renew the bloodline. Newly purchased males were not quarantined; they were treated with anthelmintics before being integrated with the rest of the herd. Specifically, the anthelmintic product “First Drench” (containing abamectin and praziquantel) was used at dosages recommended by the attending veterinarian for both sheep and goats, typically administered orally at a dosage of 2.5 ml per 10 kg body weight. Weaned lambs and goat kids received anthelmintic treatment individually and only when necessary, based on a combination of clinical signs (e.g., altered faecal consistency, reduced body condition score) and, in some cases, faecal egg count results provided by the farm veterinarian. Dairy animals did not receive any anthelmintic or anticoccidial treatment during the study period or within at least six months preceding its onset.

### Sampling procedure and parasitological methods

Twenty dairy goats and twenty dairy sheep were randomly selected for monthly rectal sampling over 12 months. The same individually numbered animals were followed throughout the study. Sampling was performed before the afternoon milking, when the animals were housed indoors; sheep and goats were separated for faecal collection and subsequent milking. Samples were stored in labelled plastic bags at 4 °C and examined the following day.

Over the course of the study, a total of 210 goat and 196 sheep faecal samples were collected. The difference from the expected maximum (240 samples per species) reflects occasional instances where faecal collection was not possible, for example, due to an empty rectum or animal handling constraints. This resulted in a small number of missing monthly samples per individual.

Nematode eggs were quantified using the Concentration McMaster method (Roepstorff & Nansen, 1998), with a sensitivity of 20 nematode eggs per gram (EPG). This method was selected due to its simplicity and cost-effectiveness, making it suitable for routine faecal egg count monitoring under farm conditions. Both the prevalence and infection intensity were evaluated as described by Bush *et al*. (1997).

Because morphological differentiation of nematode eggs to genus level is generally unreliable, all strongyle-type eggs were merged into strongylid nematodes. The remaining portions of individual faecal samples, which were not used for quantitative egg counts, were pooled and subjected to coproculture for larval development and incubated for seven days at 27 °C to obtain infective larvae (L_3_) for reliable strongylid identification. Recovered L_3_ were identified to the genus or species level according to van Wyk and Mayhew (2013). *Trichostrongylus* spp. and *Teladorsagia* spp. L_3_ were merged into one group (*Trichostrongylus*/*Teladorsagia*) due to their similar morphology, which precluded reliable differentiation.

### Statistical analysis

A power analysis was conducted before the survey to determine the effect of sample size. Differences in GIN infection intensities between goats and sheep were analysed using the Mann-Whitney test, with statistical significance set at α = 0.05. Statistical calculations were performed using Statistica 12 (StatSoft ČR, 2012). Violin plots comparing infection intensities in sheep and goats were generated using R statistical software, version 3.1.2 (R Development Core Team, 2014). In addition to the primary analyses, supplementary correlation analyses were performed separately for sheep and goats to explore potential relationships between milk production parameters and the intensity of gastrointestinal nematode infection, measured as faecal egg counts (EPG).

## Ethical Approval and/or Informed Consent

No animals were harmed during the course of the study. Faecal samples were collected non-invasively during routine handling, and all applicable national and institutional guidelines for animal welfare were strictly adhered to.

## Results

Over the 12-month monitoring period, a total of 210 samples from goats and 196 samples from sheep were analysed (see Table 2). The overall prevalence of trichostrongylid nematodes was 81 % (158/196) in sheep and 96 % (202/210) in goats. *Trichuris* eggs were also detected in goats, with an overall prevalence of 6.1 % and a maximum EPG of 180 in June. In sheep, *Trichuris* eggs were found only once, at a level of 20 EPG, also in June.

**Table 2.** Summary of faecal egg counts (EPG), prevalence, and number of samples analysed in sheep and goats throughout the 12-month monitoring period. EPG values reflect the intensity of trichostrongylid infection, while prevalence indicates the proportion of positive samples per species. The table also includes the total number of samples obtained, accounting for minor occasional sampling losses due to technical limitations.

Trichostrongylids - sheep								
prevalence (%)	median	mean	geomean	CI (%)	SD	pooled samp. (EPG)	min. EPG	max. EPG
100	1780	1747	1648	1477 – 2017	543	1340	720	2520
75	1120	990	1254	669 – 1311	687	300	0	1980
40	0	457	1103	175 – 739	602	180	0	1520
86	140	1087	326	96 – 2078	1717	460	0	5040
60	100	627	501	60 – 1193	1023	0	0	3640
80	500	856	472	305 – 1407	996	0	0	2600
93	280	692	314	170 – 1214	943	120	0	3540
94	530	607	1533	385 – 829	446	0	0	1520
80	20	375	107	0 – 872	899	140	0	3520
79	40	189	85	0 – 396	360	0	0	1260
93	700	804	643	459 – 1149	623	520	0	2140
94	1760	1639	1202	1007 – 2270	1228	580	0	3900

Trichostrongylids - goats								
prevalence (%)	median	mean	geomean	CI (%)	SD	pooled samp. (EPG)	min. EPG	max. EPG
90	180	316	214	166 – 466	319	680	0	1140
100	510	910	478	341 – 1479	1145	400	20	4380
100	900	1222	721	692 – 1752	1100	2720	80	3500
100	3500	4254	2813	2526 – 5981	3584	520	120	13740
100	5090	6555	4144	3775 – 9336	5591	540	340	20980
89	2160	2788	1992	1427 – 4148	2736	160	0	10220
100	940	1760	933	803 – 2717	1924	120	40	7380
100	730	1292	716	581 – 2003	1430	100	20	5500
100	560	734	428	336 – 1132	774	240	80	2840
94	160	531	325	228 – 834	589	480	0	1880
92	100	138	111	63 – 214	119	320	0	380
88	260	239	194	143 – 335	180	340	0	540

The seasonal dynamics of egg shedding, as depicted in Table 2, show that both species experienced fluctuations in nematode transmission throughout the year. However, these seasonal changes did not result in consistent differences in infection intensity between goats and sheep.

**Fig. 2:**
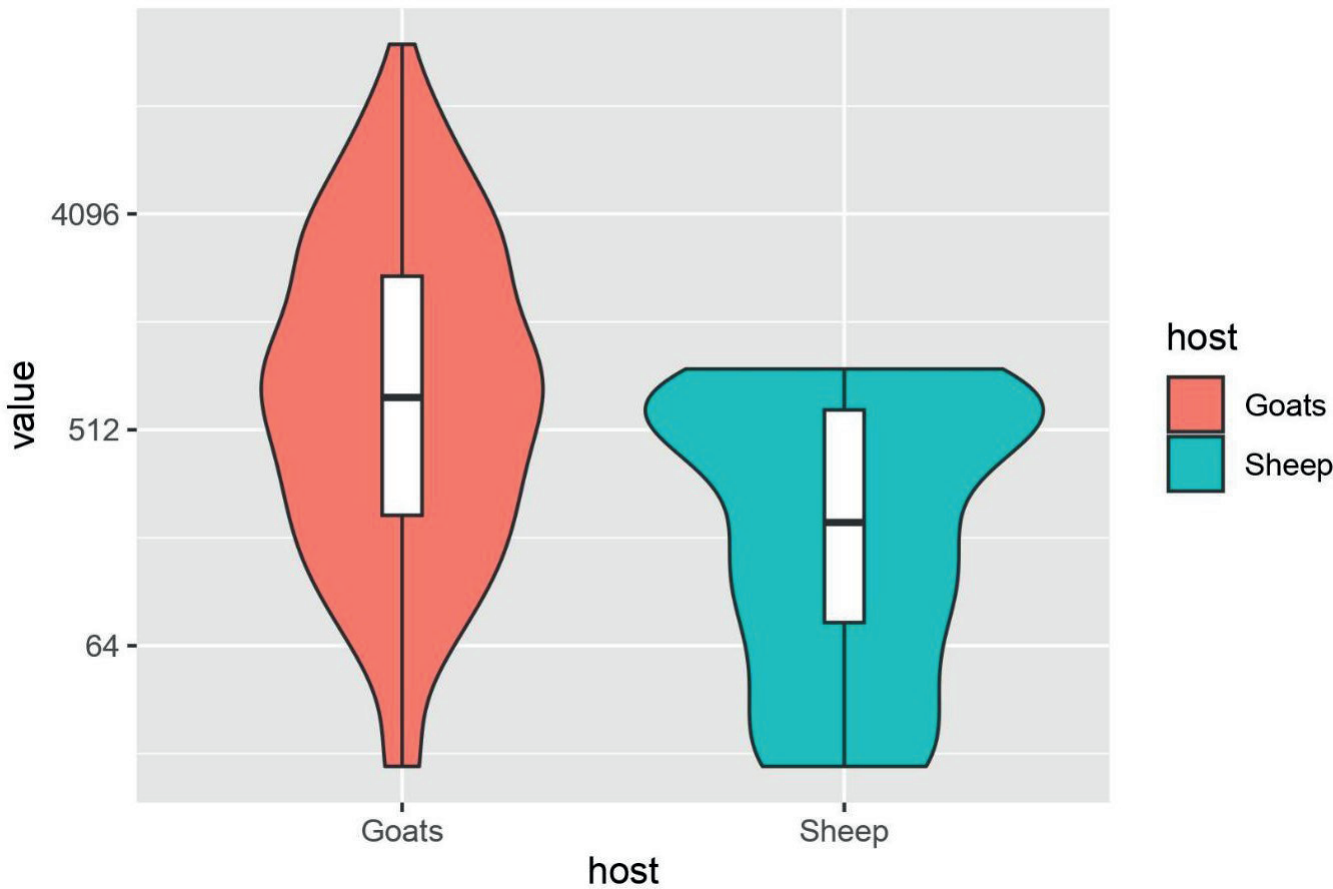
Comparison of strongylid nematode infection in sheep and goats. Data are expressed as eggs per gram of faeces (EPG) and were log-transformed for visualization. Violin plot displays the distribution, median (white dot), interquartile range (thick bar), and density. Higher median and variability were observed in goats. Differences between species were statistically evaluated using the Mann-Whitney U test (U = 24,697.5, p < 0.001).

When data from the entire 12-month period were analysed, the overall infection intensity, expressed as eggs per gram of faeces (EPG), was significantly higher in goats compared to sheep (Mann-Whitney U test, U = 24,697.5, p < 0.001). Despite comparable prevalence, goats showed greater individual variability and higher maximum EPG values (max. 20,980 EPG in June), whereas the maximum in sheep was 5,040 EPG (in May). The distribution of infection intensity is visualized in Fig. 2. Violin plots reveal that while both groups share overlapping ranges of EPG values, goats tend to exhibit higher median values and more pronounced tails, reflecting a broader distribution and a greater number of extreme cases. This confirms greater heterogeneity of infection among goats and supports the statistical findings. Differences in infection intensity between sheep and goats were statistically significant (Mann–Whitney U = 24 697.5, p < 0.001).

A more detailed monthly analysis (see Table 2) revealed significant differences in infection intensity across several months. Sheep had significantly higher EPG values in February (U = 5.0, p < 0.001), whereas goats had significantly higher EPG values in April, May, June, July, August, October, and November (all p < 0.05). No significant differences were found in March and September. These results indicate seasonal shifts in the parasite burden, with goats showing peak infection during the summer months and sheep experiencing a transient peak in late winter. Spearman’s rank correlation analysis was conducted to evaluate the relationship between the intensity of strongylid nematode infection in goats and sheep. There was a weak but statistically significant negative correlation between the two variables (R = –0.20, p = 0.0028, n = 218), suggesting a slight tendency for higher infection levels in goats to coincide with lower infection levels in sheep. However, the strength of this association was low.

*Haemonchus contortus* was the most frequently detected species and remained dominant in the goat population throughout the year. In sheep, the dominant species shifted seasonally, with *Trichostrongylus/Teladorsagia* spp. becoming more prominent during certain months. *Oesophagostomum columbianum* was detected at lower levels in both host species.

**Fig. 3.**
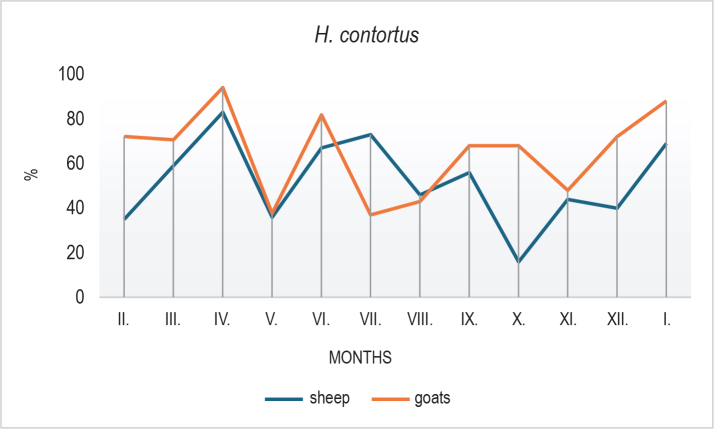
Seasonal composition of *Haemonchus contortus* larvae isolated from faecal cultures of goats and sheep over the 12-month monitoring period. *H. contortus* predominated in goats throughout the year, while in sheep, its prevalence fluctuated seasonally, with a decline in autumn and resurgence in winter months.

**Fig. 4.**
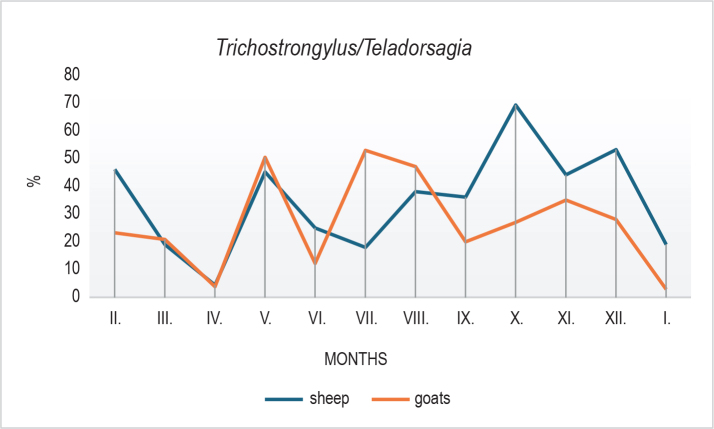
Proportion of *Trichostrongylus/Teladorsagia* spp. larvae recovered from faecal samples during the study. In goats, their abundance increased during the summer, while in sheep a marked peak was observed in October. The grouping reflects the indistinguishable morphology of L_3_ larvae from these two genera.

**Fig. 5.**
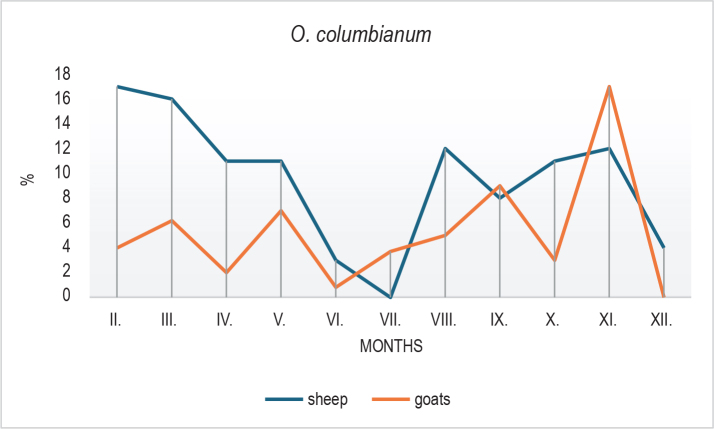
Seasonal dynamics of *Oesophagostomum columbianum* larvae identified in coprocultures from goats and sheep. A general decline was observed during the summer (June–July) in both host species, followed by fluctuating increases in the autumn and winter months.

Clear seasonal patterns were also observed in the larval composition. In sheep, *H. contortus* declined markedly in October (Fig. 3), followed by an increase in winter months, possibly reflecting microclimatic influences, larval hypobiosis, or delayed development. In goats, *Trichostrongylus*/*Teladorsagia* spp. peaked in July (Fig. 4) with a subsequent decline, while sheep showed a peak in October. *O. columbianum* (Fig. 5) was reduced in summer (June – July)in both species and showed variable increases during autumn and winter.

## Discussion

Co-grazing of sheep and goats is practiced in many temperate and Mediterranean regions, but its effect on GIN epidemiology remains debated. Goats and sheep share most GIN species, with goats often producing higher egg counts during the grazing season, which may potentially increase the infection risk for sheep (Hoste *et al*., 2008; Hoste *et al*., 2010). In this study, we compared GIN species richness and infection intensity in sheep and goats co-grazed on a conventional dairy farm.

We found no significant difference in GIN species richness between sheep and goats. In both species, *Haemonchus contortus* was the most prevalent nematode, followed by *Trichostrongylus*/*Teladorsagia* and *Oesophagostomum columbianum*. However, *H. contortus* predominated in goats throughout the year, while in sheep, *Trichostrongylus/Teladorsagia* spp. showed periodic dominance. This seasonal variation aligns with previous studies (Torina *et al*., 2004b; Hosté *et al*., 2010; Domke *et al*., 2012; Kelly *et al*., 2012; Domke *et al*., 2013; Kyriánová *et al*., 2019; Ruiz-Huidobro *et al*., 2019). Torina *et al*. (2004) highlight the complexity of parasite dynamics in mixed-species grazing systems. In addition to the dominant species of the genus *Trichostrongylus*, less abundant species were also found, of which *H. contortus* was the most common. In contrast, in goats, the authors reported only *T. circumcincta* and *T. axei* as the dominant nematodes in the abomasum; all other nematode species were less represented.

While numerous studies have examined GIN infections in sheep and goats, most of them focused on systems where the species were reared separately. Studies specifically addressing co-grazing systems remain limited. However, recent research using metabarcoding approaches has provided new insights into the dynamics of GIN infections under mixed-species grazing. Notably, Costa-Junior *et al*. (2021) and Mohammed *et al*. (2024) observed that *Haemonchus contortus* was consistently dominant in goats, whereas sheep harbored more diverse nematode communities, including *Teladorsagia* and *Trichostrongylus* species. Both studies also reported seasonal shifts in species composition, underscoring the influence of host species and seasonality on parasite epidemiology. Our results align with these findings. In goats, *Haemonchus contortus* predominated throughout the year, whereas in sheep, *Trichostrongylus* and *Teladorsagia* spp. showed periodic dominance. These patterns suggest that parasite dynamics may differ between co-grazed species, underlining the potential benefit of considering host-specific factors in parasite control. Further comparative research is needed to evaluate whether control strategies developed for single-species systems are equally effective in co-grazing contexts. This is particularly relevant considering our findings that, despite distinct seasonal peaks—such as the higher mean EPG in goats in June and in sheep in February—the overall infection pressure in both groups remained stable and of similar magnitude throughout the study period. Although individual months revealed significant differences in EPG between host species, these differences were inconsistent and likely reflect short-term environmental or physiological fluctuations. These results support the conclusion that co-grazing, as practiced in this study under a rotational pasture system and without recent anthelmintic treatment, does not increase the risk of gastrointestinal nematode infection in either sheep or goats. While species-specific responses to GIN exposure were observed, the comparable prevalence and long-term infection intensity across hosts suggest that mixed-species grazing per se is not a primary driver of elevated parasite transmission under the given conditions.

The presence of GIN infective larvae on the vegetation and their availability to the host are influenced by numerous external and internal factors. Key external factors include local climatic conditions and microclimatic conditions on the pasture. Temperature and moisture play a dominant role in the development of the trichostrongylid free-living stages (O’Connor *et al*., 2006). Interestingly, *H. contortus* was most prevalent during winter in our study, contrary to its typical ecological pattern. Possible explanations include specific microclimatic conditions, pasture management practices that affect larval survival, or the resumption of development from hypobiotic larvae. Further research is needed to clarify these mechanisms.

The large pasture area, relative to the number of animals, likely reduced larval density through host dispersal and natural mortality (Torres-Acosta & Hoste, 2008). Faecal moisture and dense vegetation may also prolong larval survival (van Dijk & Morgan, 2011). Internal factors, such as host immunity, differ between sheep and goats, with goats’ browsing behaviour potentially reducing contact with infective larvae but also limiting immune development (Houdijk *et al*., 2012). Another key factor influencing nematode prevalence is anthelmintic resistance (AR). Anthelmintic resistance, reportedly higher in goats, may further contribute to the dominance of *H. contortus* (Hoste *et al*., 2011).

Furthermore, *H. contortus* is known for its high fecundity, with females laying up to 10000 eggs daily, compared to *Trichostrongylus*/*Teladorsagia*, which produce only around 100 eggs per day. This reproductive advantage means that even at similar infection levels, *H. contortus* has a disproportionately higher impact on pasture contamination. Finally, differences in host specificity between ovine and caprine GIN lineages may play a role in infection dynamics. Studies suggest that sheep are less susceptible to caprine GIN lineages, which could further affect nematode transmission dynamics in co-grazing systems.

These findings highlight the complexity of host-parasite interactions in mixed-species grazing systems and underscore the need for further research into environmental, genetic, and management factors influencing GIN epidemiology. The cross-transmission of resistant nematodes between species, as well as between domestic and wild ruminants, has been recently documented, further emphasizing the importance of sustainable parasite control strategies (Beaumelle *et al*., 2024). Additionally, studies on anthelmintic resistance in small ruminants suggest that goats exhibit higher resistance levels, likely due to more frequent and often suboptimal treatment applications. Hoste *et al*. (2010) emphasized that improper dosing, combined with the pharmacological differences between goats and sheep, may contribute to selection pressure and subsequent shifts in nematode species composition.

During our study, no significant differences in the intensity of GIN infections were detected between sheep and goats. Similar patterns were observed in India by Singh *et al*. (2021) and Kumar *et al*. (2019), who reported comparable EPG values across both species, with infection levels strongly influenced by seasonal conditions. In contrast, Domke *et al*. (2013) observed higher EPG levels in sheep than in goats. Our study confirmed that goats shed increased numbers of eggs during the season; however, this shedding pattern was not consistent and did not appear to impact infection intensity in sheep significantly. The observed seasonal variations and differences in infection intensities between sheep and goats suggest that multiple factors, including environmental conditions, anthelmintic resistance, and host-specific immune responses, contribute to shaping the dynamics of GIN infections in mixed-grazing systems. The dominance of *H. contortus* in goats may be linked to its higher fecundity and ability to develop resistance to anthelmintics more rapidly than other nematode species. Additionally, differences in host grazing behaviour and susceptibility to specific nematode lineages could further explain the infection trends seen in this study. Future research should focus on integrating detailed environmental monitoring, genetic analysis of nematode populations, and long-term studies on anthelmintic resistance to develop effective parasite management strategies for mixed-species grazing systems.

## Conclusions

Overall, our results showed that goats consistently exhibited higher GIN infection intensities than sheep, with mean peak faecal egg counts of 1 240 EPG in June compared to 620 EPG in February in sheep. *Haemonchus contortus* predominated in goats year-round, while *Trichostrongylus*/*Teladorsagia* spp. in sheep displayed pronounced seasonal peaks, with coproculture of larvae showing a predominance of up to 60 % *Trichostrongylus*/*Teladorsagia* larvae in sheep in the autumn. These findings fulfill the study aim of characterizing species composition and infection intensity in co-grazed small ruminants, supporting the hypothesis that co-grazing does not homogenize parasite burdens between host species. The observed species-specific and seasonal patterns highlight the need for targeted, host- and season-specific parasite control strategies, as well as further research into environmental and management factors that influence GIN epidemiology in mixed-species grazing systems.
